# Electrically Self-Propelled Vehicles

**Published:** 1913-08-30

**Authors:** 


### ELECTRICALLY SELF-PROPELLED VEHICLES.

### American Experience of the Electrical Ambulance.

The two most important things required for an ambulance are speed and absence of jolting. The electrically driven ambulance presents very peculiar advantages on both these points. The principal objection to them is the cost of propulsion. Notwithstanding this, in America the electrically driven vehicle is making steady headway, both against the horse-drawn vehicle and against petrol, or, as they call it on that side, gasoline. In the case of the ambulance, however, this great objection disappears to a large extent. The ambulance is necessarily only required at intervals. It may have a great deal of work comparatively during a short period, and then not be required for a long time. But when it is working its hardest the work required from it is only a fraction of that required from the ordinary delivery van. If, therefore, the electrically driven delivery van is taking the place of horse and gasoline vans in America, it is clear that the electrical method of driving an ambulance is at least feasible.

The second important requirement, absence of jolting and general comfort of the patient, is best fulfilled by the electrically driven vehicle, in which there is no vibration such as is unfortunately necessary with the petrol engine.

In all self-propelled vehicles power has to be provided. In the horse-drawn vehicle the horse provided it; in the petrol-driven vehicle it is obtained from a supply of that spirit utilised in an engine designed for the purpose. In the electrically driven vehicle the power is supplied by electricity taken from a secondary battery—or accumulator, as it is called—similar to those used with $x$-ray apparatus. There are a good many more cells in the battery than is usual with $x$-ray apparatus, but the individual cells are usually not so large. The secondary battery, or accumulator, enables a supply of electricity to be carried about, and to be used as and when it is wanted. The battery has to be charged at uncertain periods, depending upon the amount of work the vehicle has done, just as with the battery attached to $x$-ray apparatus, and the method of charging is exactly the same. Nowadays any electricity generating station can furnish a charge for any accumulator that is brought to it at no great cost. The accumulator must be charged with continuous, not sinusoidal, currents; but all generating stations, whatever the form of current they generate for public supply, have means of converting to continuous currents whenever they are required.

With mechanically propelled vehicles there is the wide distinction that the hind wheels usually have to be made to turn in order that the vehicle may move; while with horse-drawn vehicles the wheels merely roll as the horse draws the vehicle onwards. Thus it is necessary for the power to be conveyed to what are termed the driving wheels, usually the rear wheels. With the petrol engine the power is transmitted by a system of toothed wheels, which have to be thrown in and out of mesh when the speed is changed, or when the vehicle is to be propelled backwards. In consequence of the fact that the petrol engine can only run in one direction, a clutch has to be provided to disconnect the toothed wheels from the engine when the vehicle is standing, and also when changes of speed are required. Every change of speed, every stop and restart, is accompanied by a certain amount of jerking. With the electrically driven vehicle the power is delivered to the driving wheels by electric motors, which can be turned in either direction at will, and whose speed can be varied as gradually as required by the motion of a lever at the driver's hand; so there is no jolting when hills have to be ascended or descended; nor, again, when the car has to be stopped in a press of traffic. The driver has the speed and direction of motion—forward or backward—directly under his control by simple motions of a controlling lever arranged very much in the same manner as the controlling lever of an electric tramcar. The whole of the gearing is always in mesh, in contradistinction to the petrol-driven car, where it is constantly being unmeshed and remeshed.

The accumulators in use for electrically driven vehicles have been, up till recently, those which have been on the market for some thirty-odd years, in which lead plates and lead oxides are employed. There is not much difference, except in details of manufacture, rendering the batteries more reliable and less expensive to maintain between those on the market to-day of the lead type and those introduced thirty years ago. Recently, however, Mr. Edison has introduced the accumulator upon which he has been working for so many years, in which iron and nickel are employed, and in which caustic potash is the liquid electrolyte employed in place of the dilute sulphuric acid used in the lead battery. A great many important improvements have been introduced in the Edison battery. The active material, as it is called, is all enclosed in nickel-plated steel boxes, perforated to allow the liquid to circulate, and the boxes are all held in nickel-plated steel frames. This arrangement is designed to give much-needed strength to the component parts of the battery.

The pressure of the Edison cell is very much less than that of the lead battery, which means that many more of them have to be used for any given work; but this is not an important matter. The Edison cell is still on its trial, and it is not yet absolutely certain that finality has been attained.

In The Hospital of July 26 (page 509) reference was made to the electric motors which are in use at Queen Mary's Hospital for Children, Carshalton, for carrying the meals from the central kitchen to the scattered blocks of which the institution is composed. They are designed to carry 30 cwt., and are in the form of covered vans, which are described as working very satisfactorily.

